# The Cost of Drugs

**Published:** 1905-10-07

**Authors:** 


### HOSPITAL ADMINISTRATION.

### CONSTRUCTION AND ECONOMICS.

### THE COST OF DRUGS.

A CONTRIBUTION TO HOSPITAL ECONOMICS.

By the Chief Dispenser of a Large Hospital.

(*Continued from page* 472 )

We may now conveniently turn to examine a little more closely the general statement that galenicals cannot be produced as cheaply, pound for pound, in a well-equipped pharmaceutical laboratory attached to a large institution as in that of the wholesale factories. It is obviously desirable to look at this from all sides. We must suppose that in such an institution steam is available. It is there; it must be for heating purposes. It is not generated specially for laboratory purposes as in a wholesale warehouse; it has to be there, whether you utilise it or not. We must suppose that adequate fittings and appliances, as indicated above, are provided so organised and equipped that the advent of intervals of more or less freedom are immediately utilised to the greatest profit and advantage. Much of this work is the result of processes which simply require starting and occasional tending to be perfectly done, and with labour which practically costs nothing, because those employés, as we demonstrated, have to be present in any case. The wholesale warehouseman maintains his staff for the purpose of making those very preparations—and this adds considerably to the cost of production so far as he is concerned. Then steam, an important factor in the question of cost, which is circulating in the heating coils, cannot be said to be so much an element of cost as if it were specially generated with all the costly equipment which has to be maintained exclusively for this purpose in a drug-warehouse. It is obvious that a laboratory of this kind in such an institution is at no small initial advantage. But another point in which it has an important and, to those in the know, as the expression goes, very real advantage, is the attainments and training of its employés. In nine-tenths of the wholesale houses probably only one man in each department knows the work both in its routine and ultimate requirements. It is also a fact that much of this work is consequently left in the hands of employés who are largely automatons, and certainly know little or nothing of what constitutes perfect galenicals in relation to their ultimate use by the physician or surgeon. This is no inconsiderable drawback. But leaving the matter of personnel and equipment, we may glance for a moment at the cost of raw materials. Cost of materials is of course largely conditioned by where, how, and under what stipulations purchasing is done. If you are buying raw material, and know your business, it is comparatively easy to be sure of the quality (for we must imagine this to be placed first) of supplies. They cannot cheat you with the quality of the gentian root, calumba root, strophanthus seeds, digitalis leaves; they cannot sell you maize starch at rice starch price; you cannot be cheated with rhubarb root or a colocynth at double its market value. But it is needless to multiply illustrations; they are endless. The net result is that, in the important point of quality, the person who buys the selected raw material, and makes preparations strictly by British Pharmacopœia methods, is in an assured position compared with one who buys concoctions, since in very many cases no analyst can possibly say whether or not they have been made from good materials.

Having decided for quality, and having seen the security attained by purchasing the crude drug, the conditions of purchase may claim attention. Such an institution will buy largely—hundredweights, tons—in many cases on the most favoured terms, and, when possible, from actual importers or producers. Their money will be sure and probably spot cash, or at most one month. This practically means cash in hand. Then many houses—the best of them, indeed—consider it a distinction, and rightly so, to supply such an institution, especially if it is known that materials are so keenly scrutinised; or if the hospital is associated with a large medical school, the buyer is practically on drug sale terms. The writer buys many things at less than the wholesale price, for side considerations—as distinction of getting introduced and the first-class advertisement involved, although, of course, this hardly applies to crude drugs. The direct inference is that by judicious arrangement raw materials are secured at rates much on a par with the wholesale.

Now to converge these considerations in relation to the arguments on the other side. Purchasing ready-made galenicals, largely and competitively, without the means and time for thorough examination and testing, must mean, sooner or later, a depreciation of quality. Theoretically it is all right, practically we recognise the fallacies. In the matter of cost and quality of labour the advantage lies almost wholly with those who recommend proper equipment and manufacture in the individual institutions. Finally, the broad assertion that galenicals cannot be produced as cheaply in this way as by wholesale firms is untenable. It is quite the other way. So that as matters stand in the discussion, even now the case advanced by those who are opposed to the preparation of medicaments in hospitals cannot be defended for a moment.

But there are yet other arguments which can be urged on the other side. I make bold to say that a pharmaceutical laboratory only equipped for simple compounding cannot discharge its functions in that institution when all the enormous (often costly) supplies not classifiable as galenicals, such as oils (fatty and volatile), ether, chloroform, alcohols, formaldehyde, hydrogen peroxide, chemicals of all kinds, malt, and malt and oil, etc., have to be taken on "guarantee." This blessed word, like Mesopotamia, is mainly an anodyne for anxious spirits. The writer has at one time or another found all the substances named, and a great many more, bought "guaranteed," to be wretchedly defective. Malt and oil, to give only one instance, recently bought under a guarantee of a minimum oil value of 15 per cent. was found to contain only 3 per cent.

Looking at this mere outline, who can for a moment doubt what is the proper course for the authorities of a great hospital? This is part of the work of spare moments spread over the staff, securing as it does not only great economy and confidence in the nature of the medicaments sent to the physician, but incidentally supplies at the same time a splendid training for the young pharmacist or student. In my view the efficient checking of such materials, leaving out of sight altogether the preparing of galenicals, would almost justify and demand such equipment. But there is another aspect of the results of such a view of hospital pharmaceutical equipment. There must be throughout the year a very considerable loss that might readily be obviated, such as residues containing spirit which has been used for certain purposes in operations and bacteriological work, etc. What is done with all this in a badly equipped place? It is simply thrown away. The writer has quantities of ether, alcohol, etc., used for special processes which have been recovered quite a dozen times and used again for the same purposes.

In the preceding remarks a great deal is left out which might have been introduced. For example, we might have endeavoured to compare the cost of production of certain things; but the varying cost in raw materials arising from quality makes such comparisons rather unsatisfactory—indeed, nearly impossible. But we will take one case—namely, sweet spirit of nitre. This is practically distilled at the cost of the spirit used. Now, a large institution placing an annual contract direct with distillers need not pay more than 18s. 9d. for triple distilled British grain spirit, and it is open to question if a wholesale drug-warehouse can get any better terms. This works out at about 2s. 3d. per lb. The wholesale list of a London house prices it at 3s. 1d. per lb., and a large provincial establishment quotes 3s. 4d. per lb.; but although they gave a discount of 15 to 20 per cent. there would still be an ample margin justifying its preparation. Besides, we have a freshly made, strictly B.P. preparation, which, if stored in 1-lb. blue or amber tinted bottles, completely filled, sealed, and inverted, may be kept in perfect condition to the last drop—a very different thing from the insipid stuff that just manages to scramble past the legal standard when first bought in. A second example may be taken in the so-called aerated waters. Making every deduction dictated by business considerations, these all over may be made of very satisfactory quality for the fraction of a penny less than a shilling per dozen syphons, that is to say, less than a penny per syphon. Lastly, where the laboratory is poorly equipped small occasional pharmaceutical requirements which could not be placed on contract sheets have to be bought in as they arise, and for such a contracting house does not forget to charge. On these side lines no inconsiderable loss must annually occur.

In conclusion, I trust the statement will appear reasonable that in a properly equipped laboratory galenicals can be prepared more cheaply than in the wholesale factory, which has not only its actual operative staff to maintain, but all the necessary departments—clerks, travellers, means of distribution, etc., as well as interest on invested capital and sundry liabilities to reckon with, such as bad debts. Further than this, the failure to provide for the proper undertaking of this work in an institution such as we have been contemplating may be regarded as an economical error and betrays a want of knowledge of what the functions of such a department are and what it might accomplish.

But the same observations, with some discrimination, apply to much smaller institutions than the hypothetical one we have been keeping in view. There can be no return to the haphazard methods of the past. With the advent of asepsis and sterilisation in surgery came applications in practical pharmacy and dispensing which may not be ignored if it be the aim and function of that department to realise the wishes of the physician or surgeon, and this, it appears to me, can only be done efficiently and economically on the lines I have endeavoured to lay down; and, with a single eye to the interests of the institution, proper equipment, and assiduous management, a great economy is assured.

